# Influence of complement protein C1q or complement receptor C5aR1 on gut microbiota composition in wildtype and Alzheimer’s mouse models

**DOI:** 10.1186/s12974-023-02885-9

**Published:** 2023-09-19

**Authors:** Tiffany J. Petrisko, Matthew Gargus, Shu-Hui Chu, Purnika Selvan, Katrine L. Whiteson, Andrea J. Tenner

**Affiliations:** 1 Department of Molecular Biology & Biochemistry, University of California, Irvine, 3205 McGaugh Hall, Irvine, CA 92697-3900 USA; 2 Department of Neurobiology and Behavior, University of California Irvine, Irvine, CA USA; 3 Department of Pathology and Laboratory Medicine, University of California, Irvine, School of Medicine, Irvine, CA USA

**Keywords:** Alzheimer’s disease, Microbiome, Complement, C1q, C5aR1, Neuroprotection

## Abstract

The contribution of the gut microbiome to neuroinflammation, cognition, and Alzheimer’s disease progression has been highlighted over the past few years. Additionally, inhibition of various components of the complement system has repeatedly been demonstrated to reduce neuroinflammation and improve cognitive performance in AD mouse models. Whether the deletion of these complement components is associated with distinct microbiome composition, which could impact neuroinflammation and cognitive performance in mouse models has not yet been examined. Here, we provide a comprehensive analysis of conditional and constitutive knockouts, pharmacological inhibitors, and various housing paradigms for the animal models and wild-type controls at various ages. We aimed to determine the impact of C1q or C5aR1 inhibition on the microbiome in the Arctic and Tg2576 mouse models of AD, which develop amyloid plaques at different ages and locations. Analysis of fecal samples from WT and Arctic mice following global deletion of C1q demonstrated significant alterations to the microbiomes of Arctic but not WT mice, with substantial differences in abundances of *Erysipelotrichales*, *Clostridiales* and *Alistipes.* While no differences in microbiome diversity were detected between cohoused wildtype and Arctic mice with or without the constitutive deletion of the downstream complement receptor, C5aR1, a difference was detected between the C5aR1 sufficient (WT and Arctic) and deficient (C5ar1KO and ArcticC5aR1KO) mice, when the mice were housed segregated by C5aR1 genotype. However, cohousing of C5aR1 sufficient and deficient wildtype and Arctic mice resulted in a convergence of the microbiomes and equalized abundances of each identified order and genus across all genotypes. Similarly, pharmacologic treatment with the C5aR1 antagonist, PMX205, beginning at the onset of beta-amyloid plaque deposition in the Arctic and Tg2576 mice, demonstrated no impact of C5aR1 inhibition on the microbiome. This study demonstrates the importance of C1q in microbiota homeostasis in neurodegenerative disease. In addition, while demonstrating that constitutive deletion of C5aR1 can significantly alter the composition of the fecal microbiome, these differences are not present when C5aR1-deficient mice are cohoused with C5aR1-sufficient animals with or without the AD phenotype and suggests limited if any contribution of the microbiome to the previously observed prevention of cognitive and neuronal loss in the C5aR1-deficient AD models.

**Supplementary Information:**

The online version contains supplementary material available at 10.1186/s12974-023-02885-9.

## Introduction

Alzheimer’s disease (AD) is a neurodegenerative disorder affecting approximately 40 million people worldwide that contributes to 60–80% of all dementia cases. Cognitive loss follows AD’s hallmark pathology of extracellular fibrillar beta-amyloid (fAβ) plaques and neurofibrillary tangles comprised hyperphosphorylated tau, loss of synapses and neurons, as well as chronic neuroinflammation [1]. The complement system is a critical part of the innate immune system that tags and removes cellular debris and pathogens in the periphery and the central nervous system, and also plays a role in sculpting synapses and thus fine-tuning neural circuits (reviewed in [2–4]). In the AD brain, complement components including C1q, C3, and C4 co-localize with fibrillar amyloid plaques and C1q and C3 fragments tag synapses promoting engulfment by microglia [5]. Furthermore, inhibition of the complement system at various points of the complement cascade has resulted in a suppression of cognitive decline in both aging and AD models (reviewed in [2]).

Growing evidence of gut dysbiosis in AD patients [6, 7] and animal models, including the 5xFAD, APP/PS1, and Tg2576 mouse models [8–11], demonstrates the importance of, and need for, further investigations into the role of the microbiome in AD. While the gut–brain axis has long been acknowledged, recent years have seen a plethora of new investigations to determine the role of the microbiome on brain health and behavior, due in part to the continued technological advancements of metagenomics [12–14]. Communication from the gut to the central nervous system occurs through several mechanisms. Firstly, production of the neurotransmitters γ-aminobutyric acid (GABA) and serotonin (5-HT) by gut bacteria allow for modulation of enteric neural pathways (i.e., vagus nerve) [14, 15]. Secondly, microbial-derived metabolites, such as short chain fatty acids (SCFAs) [16] or tryptophan metabolites, can act directly on intestinal cells or be transported across the intestinal barrier for systemic distribution and potential entry to the brain where they can reinforce blood–brain barrier integrity, reduce inflammatory signaling, and modulate neurotrophic factors [17–19]. Thirdly, gut bacteria may activate the immune system, particularly the innate immune system, resulting in increased systemic inflammation, which can induce a leaky intestinal barrier [20, 21]. It is likely through a combination of these mechanisms [9, 22], along with age-related reductions in both gut intestinal barrier [23] and blood–brain barrier integrity [24], that the microbiome modulates the inflammatory environment in the brain, thereby affecting cognition and behavior.

Soluble complement proteins are produced in the liver and then are constitutively present in circulation. Several of these proteins are proenzymes that circulate in an inactive form until activated by an external or intrinsic danger signal, triggering the production of downstream activation cleavage products. Furthermore, complement proteins (C3, Factor B) and receptors (C3aR, C5aR) can be expressed in the healthy intestinal mucosal and epithelial tissue [25, 26], and all components can ultimately be induced in immune cells, such as resident macrophages found along the length of the intestines [27]. As such, it is no surprise that the complement system, in particular the epithelial production of complement proteins, has been identified as a key regulator of homeostasis in the gut microbiome following various perturbations such as diet, drug, and/or immune challenge [28]. Conversely, ablation of complement proteins or receptors impacts microbial colonization of the skin in C5aR1 knockouts (KO) [29] and of the intestine in C3KO [30]. Importantly, a study by Zysset-Burri et al. [30], which examined the microbiota in patients with age-related macular degeneration (AMD), showed a correlation between the microbiota and single nucleotide polymorphisms in genes encoding parts of the complement system. Critically, C3KO mice demonstrated a unique microbiome composition compared to WT mice, and similar taxonomic features that distinguished these two groups were also observed in humans with AMD compared to those without AMD.

Previously, our lab has demonstrated that genetic ablation of C1q [31] or C5aR1 [32, 33], as well as pharmacological inhibition with the C5aR1 inhibitor PMX205 [34, 35], reduces neuroinflammation and/or pathology in AD models and rescues cognitive decline. To assess whether changes in pathology and behavior in AD model mice are at least partly mediated by the gut microbiome, this study combined the examination of the AD microbiome with genetic and pharmacological inhibition at different points in the complement pathway, namely C1q and C5aR1.

## Methods

### Animals

All animal experimental procedures were reviewed and approved by the University of California at Irvine Institutional Animal Care and Use Committee and conducted in compliance with the published guidelines in the NIH Guide for the Care and Use of Laboratory Animals. All mice were bred at UCI and housed under a 12-h light/dark cycle with ad libitum access to food and water and group housed unless otherwise stated. All animals were housed in the same vivarium room, which was limited to only animals under investigation by the Tenner lab. All animals were housed in standard cages with sawdust bedding. Arctic (Arc) mice carry the human APP transgene with the Indiana (V717F), Swedish (K670N/M671L), and Arctic (E22G) mutations (under the control of the platelet-derived growth factor-ß promoter) on the C57BL/6 background, resulting in the production of amyloid plaques as early as 2–3 months of age [36]. C5aR1 knockout mice generated by targeted deletion of the C5a receptor gene [37] were crossed with Arctic^+/−^ mice to produce Arctic mice lacking C5aR1 (Arc C5aR1KO) and wild-type littermate mice lacking the C5a receptor (C5aR1KO). The term C5aR1-sufficient and the abbreviation C5aR1+ will be used to denote WT or Arctic littermates expressing normal levels of C5aR1. *C1qa*^*FL/FL*^ mice were crossed to B6.129-*Gt(ROSA)26Sor*^*tm1(cre/ERT2)Tyj*^/J (Jackson, stock #008463), here designated *Rosa26*^*CreERT2*^ mice, which ubiquitously express the Cre-ERT2 fusion protein to generate *C1qa*^*FL/FL*^* Rosa26*^*CreERT*^ mice. These mice were then crossed to Arctic^+/−^ mice to generate Arc and WT animals with or without the *Rosa26*^*CreERT2*^ gene (presence/absence of *Rosa26*^*CreERT2*^ gene will be designated as Cre+ or Cre−, respectively, in results and figures). Both males and females were used in all Arctic animal experiments. Tg2576 mice, developed by Hsiao [38], overexpress the mutant 695 isoform of the human APP gene with the double mutation KM670/671NL (Swedish) under the control of the hamster prion promoter on a B6/SJL genetic background. WT (B6/SJL) littermates were used as control mice. Tg2576 hemizygous mice develop cortical amyloid plaques by 11–13 months of age [38]. As female mice display more significant amyloid plaque deposition, only female mice were used in Tg2576 experiments.

### Tamoxifen treatment

Tamoxifen (T5648; Sigma, St. Louis, MO) was dissolved in 5% ethanol/corn oil at final concentration of 50 mg/ml. Six-month-old Arc and WT *C1qa*^*FL/FL*^ mice with and without the *Rosa26*^*CreERT2*^ transgene were treated with tamoxifen at 0.2 mg/g body weight or vehicle control (corn oil) by oral gavage once a day for 5 consecutive days [39]. Animals were bled, perfused and tissue harvested at 12.5 months of age, 6.5 months after tamoxifen treatment as detailed below.

### Fecal collection

Fecal pellets were collected from separately housed C5aR1+ /C5aR1KO mice with or without the Arctic gene at 33, 37, and 40 weeks (10 months) of age, while fecal samples were collected for cohoused C5aR1-sufficient and C5aR1KO mice with or without the Arctic gene at 5 and 10 months of age. Fecal pellets for *C1qa*^*FL/FL*^ mice with or without the Arctic gene were collected when animals were at 11–12 months of age. Fecal pellets were collected from PMX205-treated Arctic animals at 2.7 and 5 months of age (2 and 12 weeks post-treatment), while PMX205-treated Tg2576 mice fecal pellets were collected at 12 and 15 months of age (prior to treatment and 12 weeks post-treatment). Fresh fecal pellets were collected in the early afternoon directly from each mouse by holding the mouse in one hand, during which the mouse can defecate directly into sterile 1.5-ml microcentrifuge tube held underneath the animal by the other hand. All samples were stored at − 80 °C until DNA extraction.

### PMX205 treatment

The C5aR1 inhibitor, PMX205, provided by Dr. Ian Campbell, Teva Pharmaceuticals, West Chester, PA, was administered in the drinking water (20 μg/ml, equivalent to ~ 3–8 mg/kg/day), based on previous results from our lab [34, 35]. Arctic and C57BL/6 WT littermates began treatment with PMX205 or vehicle (Water) at 2.5 months of age, while Tg2576 and WT B6/SJL females began treatment at 12 months of age, immediately prior to the onset of amyloid pathology in the respective models. Treatment lasted for 12 weeks in both models. All mice were singly housed and had free access to the drinking water for the duration of the treatment. Mouse weight and volume of PMX205/vehicle consumed by each mouse were measured weekly. Mice receiving only water are denoted as WT-H_2_O, Arc-H_2_O or Tg2576-H_2_O while animals receiving PMX205 are denoted as WT-PMX, Arc-PMX or Tg2576-PMX.

### DNA extraction

For the C1qa^FL/FL^ and separately housed C5aR1KO mouse studies, DNA was isolated from ~ 50 mg of fecal samples stored at − 80 °C using the ZymoBIOMICS®-DNA Miniprep Kit (Zymo Research, Irvine, CA). The purified DNA was then eluted using a low-concentration salt solution. For all remaining studies, 10% weight/volume of DNA/RNA Shield Solution (R1200-50) was added to fecal samples and DNA extraction was performed by Zymo Research, using the ZymoBIOMICS 96 MagBead DNA Kit.

### Sequencing

All sequencing was performed on an Illumina Miseq at the Genomics High-Throughput Facility at the University of California, Irvine. For the C1q ^FL/FL^ and separately housed C5aR1KO studies, gut bacterial community composition was characterized using the V4–V5 region of the 16S ribosomal RNA (rRNA) gene using the 515F-926R primer pair from the Earth Microbiome Project [40]. After demultiplexing in Qiime2, the mean reads per sample was 95,660 and the mean quality filtered reads per sample was 53,053 reads following quality trimming with DADA2. Taxonomic classification was performed by aligning sequences to the RDP 18 data set (https://benjjneb.github.io/dada2/training.html) to generate amplicon sequencing variants (ASVs) [41]. Sample reads were then rarefied to 10,500 for downstream analysis. In the remaining studies, the fecal bacterial DNA was amplified using the V3–V4 Illumina 16S primer [42]. Sequencing was performed on an Illumina Miseq at the Genomics High-Throughput Facility at the University of California, Irvine in two sets. After demultiplexing in Qiime2, the mean reads per sample for cohoused animals was 79,656 and the mean quality filtered reads per sample was 43,555 reads following trimming and taxonomic assignment as described above. Sample reads were then rarefied to 30,000 reads for downstream analysis. For all PMX studies, the mean reads per sample was 153,489 reads with an average mean quality filtered reads per sample of 64,361. PMX samples were then rarefied to 47,000 reads.

### Microbiome analysis

Statistical analysis, including rarefication, was done using R-Studio (R 3.6.2). Alpha diversity, nonmetric multidimensional scaling (NMDS), permutational multivariate analysis of variance (PERMANOVA), and beta dispersion were computed using the Vegan package [43]. The alpha diversity was determined by the Shannon Diversity Index. A two-way analysis of variance (ANOVA) followed by Tukey’s post hoc analysis was utilized to compare genotypes with or without the deletion of complement proteins or genotype and treatment combinations (Additional file 6: Table S1), whereas a three-way ANOVA was utilized to incorporate the factor of age or time post-treatment (three-way ANOVA results are available in Additional files). Student’s T-tests were used to determine significance between two groups. In cases comparing values from cohorts with different numbers, *t*-tests assuming equal variance were used. In cases where fecal samples were obtained at two different time points, all analyses were limited to animals for which sequencing data were available for both time points. In these scenarios, paired *t*-tests were used when comparing the same groups at two different times. NMDS and PERMANOVA were applied to the Bray–Curtis Dissimilarity Matrix and all datasets were checked to confirm homogeneity of variances using the betadisper function. The PERMANOVA was performed using the Adonis2 function set to 999 permutations for all analyses. For C1q inducible knockout studies using tamoxifen, we used the following formula: Genotype * Treatment + Cage ID. The formula for studies involving the constitutive deletion of C5aR1 was: Genotype * Complement Status + Cage ID, where genotype refers to the presence/absence of the APP transgene and complement status refers to the presence or absence of the C5aR1 gene. For Arctic PMX205 the PERMANOVA formula was: Genotype * Treatment. Pairwise Adonis was then used to determine which groups were significantly different from one another. For this analysis, genotype and complement status or genotype and treatment were merged into a single factor [44]. These results, as well as beta-diversity results addressing the impact of age/treatment duration as variables are available corresponding Additional files. Principal coordinate analysis (PCoA) was performed on the Bray–Curtis dissimilarities between samples using the cmd scale function in the stats package, with results visualized with ggplot2 [45] with 95% confidence intervals graphed using ggplot’s stat ellipse function, based on the car (companion to applied regression) package [46, 47].

## Results

### Deletion of C1q alters the microbiome in Arctic but not WT animals

**Fig. 1:**
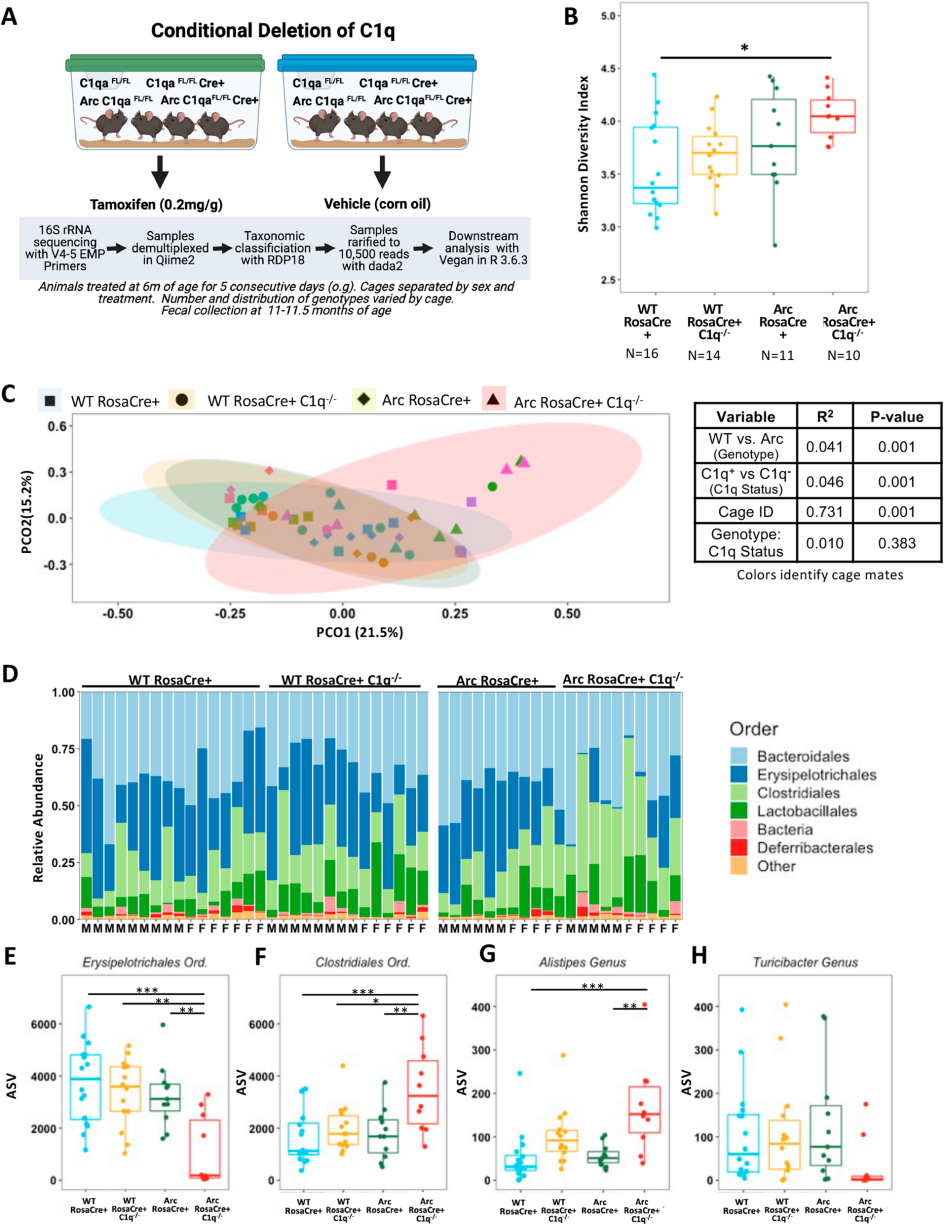
Adult deletion of C1q significantly alters Arctic but not WT microbiome. **A** Experimental overview of study design for adult deletion of C1q. **B** Alpha diversity is significantly higher in Arc C1q^−/−^ animals compared to WT C1q^−/−^ animals at 11–11.5 months of age. **C** Beta-diversity by Bray–Curtis dissimilarity plotted as PCoA demonstrates deletion of C1q^−/−^ significantly altered the microbiome of Arctic mice (green vs pink). Symbol color represents individual cages. **D** Order level taxa bar plots of RosaCre+ WT and Arctic animals with and without C1q. The number of amplicon sequence variants (ASV) for the orders *Erysipelotrichales *(**E**) and *Clostridiales *(**F**) and the genera *Alistipes* (of the order *Bacteroidales*) (**G**) and *Turicibacter* (of the order *Erysipelotrichales*) (**H)**. *p < 0.05; **p < 0.01; ***p < 0.001. ANOVA statistics are available in Additional file 6: Table S1 while beta-diversity PERMANOVA can be found in Additional file 7: Table S2. Additional details are available in Additional file 2

To investigate how deletion of the C1q component may influence the bacterial community of the gut in wildtype and AD model mice, mice with a floxed C1qa gene were crossed to the RosaCre^ERT2^ in WT or Arc mice to produce a tamoxifen-inducible C1qa deletion (Fig. 1A). Mice were group housed, often with 3 or more genotypes of varying ratios together in one cage. Males and females were never housed together. At 6 months of age, a subset of mice were given tamoxifen to induce deletion of C1qa, and thus eliminate C1q protein production in those mice containing RosaCre^ERT2^ (now referred to as RosaCre+). Vehicle- and tamoxifen-treated mice were always segregated and housed by treatment. Absence of C1q after tamoxifen in WTRosaCre+ and ArcRosaCre+ treatment was verified in plasma and hippocampus by western blot and immunohistochemistry (Methodology can be found in Additional File 5: Supplemental Methods while results are presented in Additional file 8: Fig. S1A–C). Importantly, C1q deletion in this model did not alter hippocampal or cortical amyloid plaque burden (Additional file 8: Fig. S1D, E).

The incorporation of RosaCre+ did not alter the alpha diversity, a measure of how diverse and abundant the species of microbes are in a system, or beta-diversity, a measure of similarity in species diversity between samples, when directly comparing the microbial compositions of the vehicle-treated WT and Arc mice (Additional file 8: Fig. S2). Furthermore, there was no difference in alpha or beta-diversity due to tamoxifen treatment in the WT or Arctic C1q floxed mice lacking RosaCre (Additional file 8: Fig. S3).

Importantly, treatment with vehicle or tamoxifen did not result in any significant differences in the alpha or beta diversities by Shannon Diversity Index and Bray–Curtis Dissimilarity, respectively (Additional file 8: Fig. S4A, B), in WT RosaCre+ mice, demonstrating that global deletion of the C1q gene in adult wild-type mice does not have a distinguishable impact on gut microbial diversity. Although there was no significant difference in alpha diversity (Shannon Diversity Index) between vehicle and tamoxifen-treated ArcRosaCre+ mice (Additional file 8: Fig. S4C), Bray–Curtis Dissimilarity (PERMANOVA) showed a significant attribution of 11% of the microbiome variance (*R*^2^ = 0.11, *p* < 0.009) to the deletion of the C1q gene in the Arctic mice (Additional file 8: Fig. S4D). This indicates that deletion of C1q alters the composition of the AD microbiome in ArcRosaCre+ mice.

We then addressed whether the gut microbes were different in WTRosaCre+ and ArcRosaCre+ with and without C1q present. Arctic animals were found to have an increased alpha diversity compared to their WT counterparts, regardless of the C1q status (Additional file 8: Fig. S5A). However, when alpha diversity was analyzed by two-way ANOVA (Fig. 1B), post hoc comparisons demonstrated alpha diversity was significantly increased only in the Arctic mice that had C1q deleted (ArcRosaCre+ C1q^−/−^) at 6 months of age when compared to wild-type vehicle-treated mice (WTRosaCre+).

Differences in beta-diversity are reflected in the PCoA plot, in which Arc RosaCre+ C1q^−/−^ animals demonstrated less overlapping of 95% confident interval ellipses compared to all other groups, indicating these mice contained a unique subset of microbes (Fig. 1C; Additional file 7: Table S2). Beta-diversity demonstrated differences due to the presence of the Arctic transgene (i.e., genotype; *R*^2^ = 0.041, *p* = 0.001) and by the presence/absence of C1q (*R*^2^ = 0.046, *p* = 0.001; Additional file 8: Fig S5B) but not by the interaction of genotype and presence/absence of C1q (*R*^2^ = 0.010, *p* = 0.383). Unsurprisingly, housing had the greatest influence, contributing over 73% of the variance (*R*^2^ = 0.731, *p* = 0.001). No interaction effect of sex with genotype and/or treatment was observed (data not shown), consistent with previous pathological data on the Arc mouse model [33]; however, a sex-dependent response may be visible with a larger sample size. An important point to note is that many of the variables (i.e., cage ID, genotype and treatment) used in this comparison were intricately connected as: (1) littermates of the same sex were often, but not always, housed together; (2) males and females were always housed separately; and (3) cages were separated according to treatment.

To determine what microbes may be responsible for the distinct variation of beta-diversity observed between the groups, taxa bar plots were generated at the order level (Fig. 1D), which identified the orders of *Erysipelotrichales* and *Clostridiales* as the most abundant across the genotypes. The number of *Erysipelotrichales* ASVs was significantly diminished in ArcRosaCre+ C1q^−/−^ mice, compared to all other groups, while the *Clostridiales* was significantly increased in Arc RosaCre+ C1q^−/−^ (Fig. 1E, F), again demonstrating an impact of C1q deletion in Arc but not WT mice. Previous research has identified increases in the genera *Alistipes* (of the order *Bacteroidales*) and decreases in *Turicibacter* (of the order *Erysipelotrichales*) in the fecal microbiome of AD patients compared to healthy controls [6]. While no differences were observed in *Alistipes* between WT and Arc C1q-sufficient mice, deletion of C1q in Arc animals did result in a significant increase of *Alistipes* compared to C1q-sufficient WT (*p* < 0.001) and Arc (*p* < 0.01) animals (Fig. 1G), suggesting that the deletion of C1q induces change in the AD, but not wild-type microbiota, despite the variance that can be attributed to cage effects. Although most Arc RosaCre+ C1q^−/−^ animals demonstrated low levels of *Turicibacter* (Fig. 1H), no significant differences in the abundance of *Turicibacter* were observed*.* All main figure statistics are available in Additional file 6: Tables S1 and Additional file 7: Table S2 with additional information available in Additional file 1.

A highly similar, but less robust, pattern of these results was observed when comparing only tamoxifen-treated WTC1q^FL/FL^ and ArcC1q^FL/FL^ mice with and without RosaCre (Additional file 8: Fig. S6), confirming that the observed changes in alpha and beta-diversity of the ArcRosaCre+ mice are due to the loss of C1q and not an effect of housing or tamoxifen itself (Additional file 8: Fig. S6A, B). No interaction effect of sex with genotype and/or treatment was observed (data not shown), the slight differences between these results (all animals treated with tamoxifen (Additional file 8: Fig. S6)), and those where all mice were RosaCre+ but treated with vehicle or tamoxifen in separate cages (Fig. 1), can likely be attributed to the cohousing of the mice treated with tamoxifen, resulting in more similar microbiomes between cagemates. Overall, this analysis reveals that the adult deletion of C1q has a subtle, but statistically significant, impact on the microbiome in the Arctic AD model.

### Separately housed C5aR1-deficient and C5aR1-sufficient mice have significantly different gut microbiomes

To determine how deletion of C5aR1 impacts the microbiome, and what microbial species may be driving any differences in the microbial communities, constitutive C5aR1 knockouts (C5aR1KO) were generated with both C7BL/6J (WT) and Arctic (Arc) mice. WT and Arc C5aR1-sufficient mice were housed together, separately from cohoused WTC5aR1KO and ArcC5aR1KO mice. Male and female mice were housed separately.

**Fig. 2:**
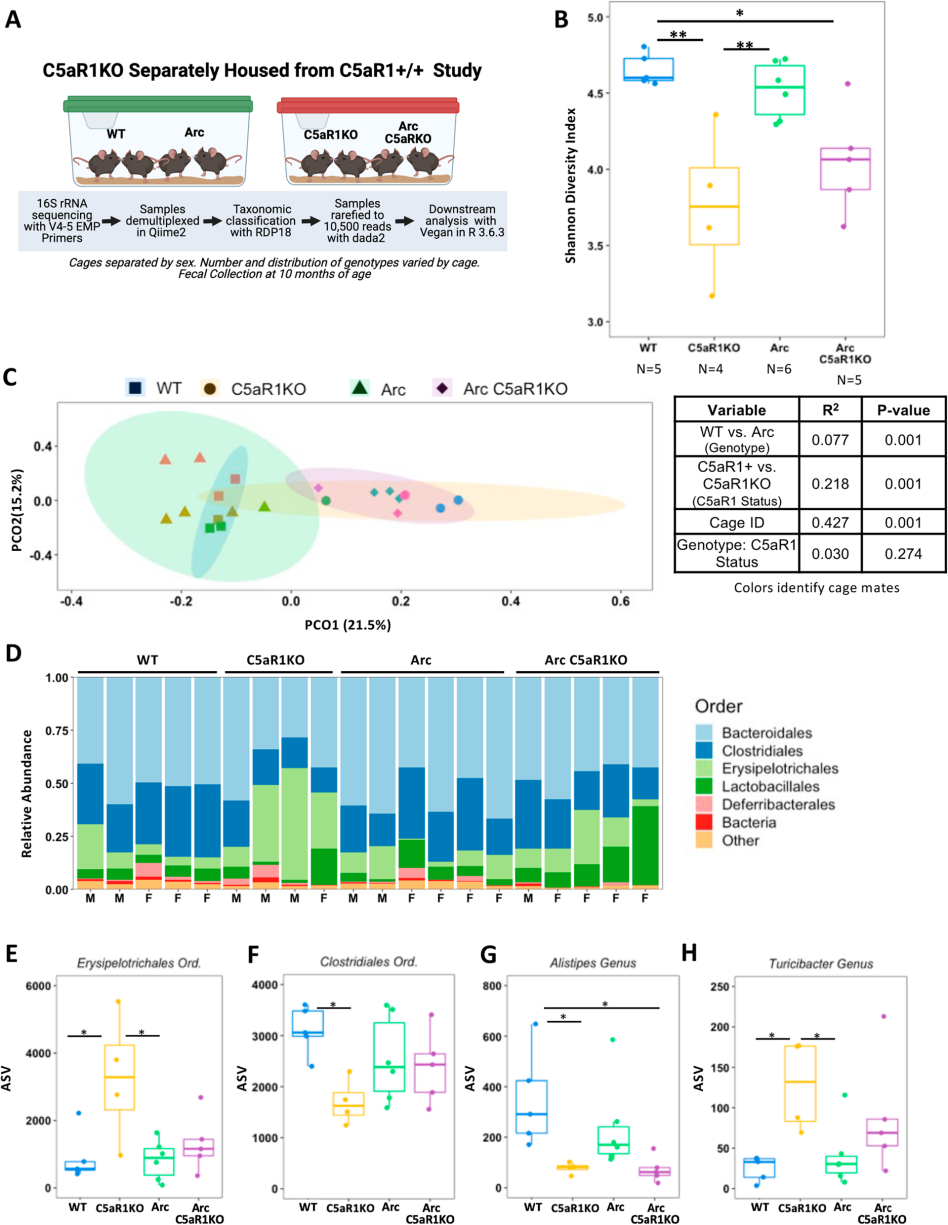
Constitutive deletion of C5aR1 significantly alters microbiome of WT and Arc mice when housed separately from their C5aR1-sufficient counterparts. **A** Experimental overview of separately housed C5aR1 sufficient and deficient animals. **B** Alpha diversity of fecal samples at 10 months of age by the Shannon Diversity Index when mice with and without C5aR1 are housed separately. **C** Beta-diversity by Bray–Curtis Dissimilarity of WT and Arctic mice with their corresponding C5aR1KO illustrates the difference in fecal microbiota composition between C5aR1KO and C5aR1-sufficient mice, with corresponding PERMOVA results to the right***.*** Symbol color represents individual cages. **D** Taxa bar plot of the 6 most abundant orders. **E–H** The number of amplicon sequence variants (ASV) for the orders *Erysipelotrichales *(**E**) and *Clostridiales *(**F**) and the genera *Alistipes* (**G**) and (**H**). *p < 0.05; **p < 0.01; ***p < 0.00. ANOVA statistics are available in Additional file 6: Table S1 while beta-diversity PERMANOVA can be found in Additional file 7: Table S2. Additional details are available in Additional file 2

The number and diversity of microbial species within an animal of each genotype (alpha diversity) was determined by computing the Shannon Diversity Index of the fecal samples. When assayed on the same day at 40 weeks of age (Fig. 2A), a significant difference between the C5aR1-sufficient (defined as WT and/or Arctic mice containing normal expression of C5aR1) and C5aR1-deficient mice, independent of the Arctic APP transgene, was observed (Fig. 2B). This result is confirmed by post hoc analysis demonstrating WTC5aR1KO mice had a significantly lower alpha diversity than their WTC5aR1+ counterparts (*p* < 0.01), with a similar trend between ArcC5aR1KO and ArcC5aR1+ animals (*p* = 0.085, Fig. 2B).

The Bray–Curtis Dissimilarity matrix was used to produce a beta-diversity PCoA plot of these mice. Permutational multivariate analysis of variance (PERMANOVA) analysis revealed a significant effect of the Arc transgene (*R*^2^ = 0.077, *p* < 0.001), and the presence vs. absence of C5aR1 (*R*^2^ = 0.218, *p* < 0.001), but not when comparing the Arc genotype with or without C5aR1 (*R*^2^ = 0.030, *p* = 0.274. Fig. 2C), indicating that the C5aR1 deletion results in significant differences regardless of the presence of absence of the Arc transgene (Additional file 7: Table S2). However, mice lacking C5aR1 were housed separately from those containing the C5aR1 gene, and the analysis showed that the cage effects accounted for the 43% of the observed variation (*R*^2^ = 0.427, *p* < 0.001). These differences were further defined by relative abundance plots at the order level that revealed *Bacteroidales, Clostridiales,* and *Erysipelotrichales* to be the top 3 most abundant orders (Fig. 2D).

The abundance of *Erysipelotrichales* (Fig. 2E) was increased in WTC5aR1KO mice compared to WTC5aR1+ mice (*p* < 0.05). *Clostridiales* abundances (Fig. 2F) were also influenced by the presence or absence of C5aR1; however, this effect was specifically limited to WT mice, with C5aR1KO mice having reduced *Clostridiales* abundance when compared to WTC5aR1+ mice (*p* < 0.05). Deletion of C5aR1 in both WT and Arc animals significantly reduced the levels of *Alistipes* in Arc compared to WT animals (Fig. 2G), but not in WTC5aR1KO compared to ArcC5aR1KO animals. WTC5aRKO animals had significantly higher levels of *Turicibacter* compared to both WT and Arc animals (Fig. 2H); a similar trend, though not statistically significant, was seen when Arc was compared to the ArcC5aR1KO. All two-way ANOVA statistics are available in Additional file 6: Table S1 with additional information available in Additional file 2. Together, these results demonstrate that the deletion of C5aR1 has a significant impact on the microbiome in both healthy and AD mice when C5aR1KO mice are housed apart from their C5aR1-sufficient counterparts.

### Cohousing of C5aR1KO and C5aR1-sufficient animals prevents microbiome divergence

**Fig. 3:**
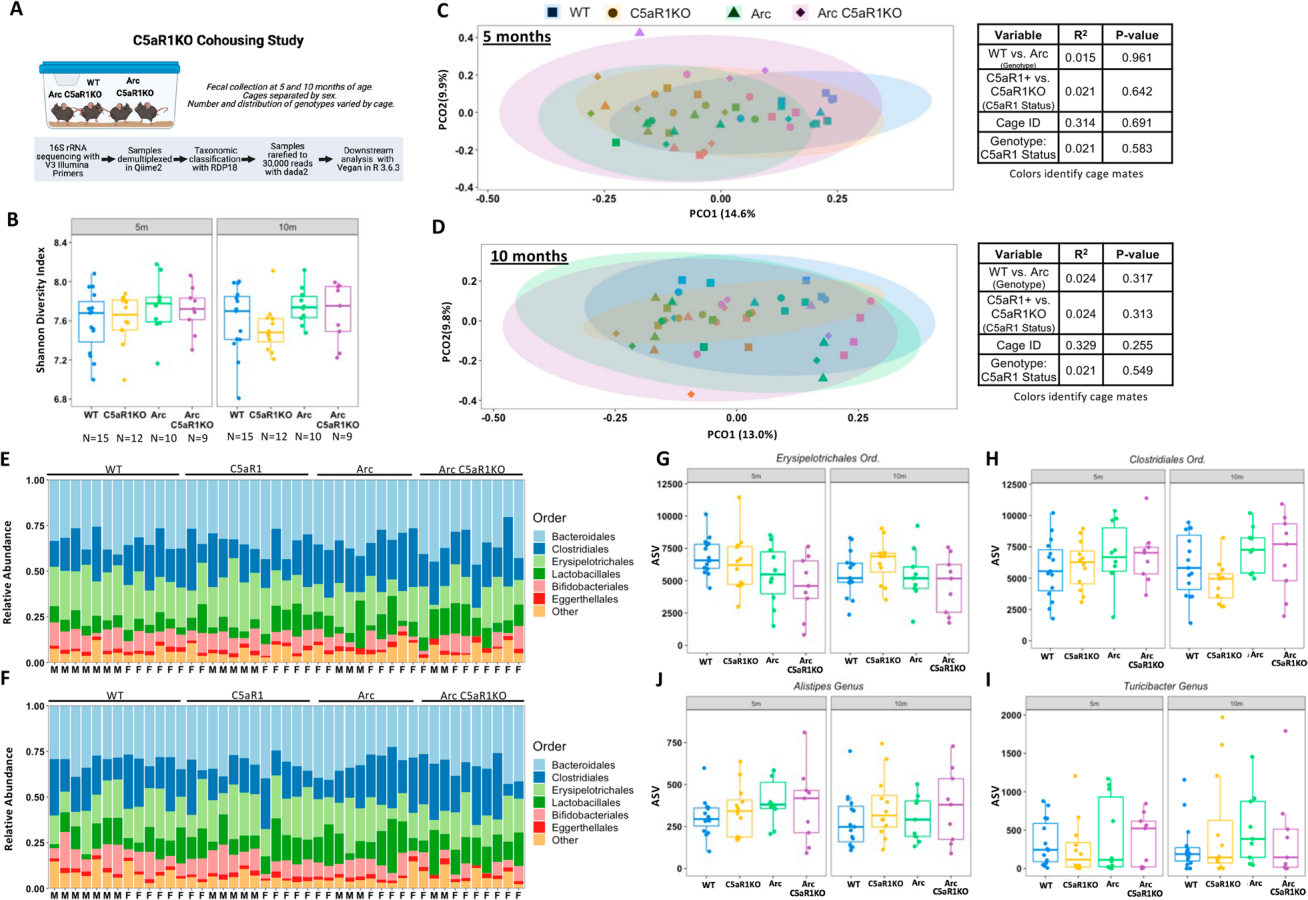
Cohousing of C5aR1-sufficient and C5aR1KO mice results in microbiome convergence at 5 and 10 months of age. **A** Schematic of C5aR1KO cohousing study design. **B** Alpha diversity at 5 months and 10 months. Beta-diversity, as measured by Bray–Curtis dissimilarities at 5 (**C**) or 10 (**D**) months are presented as principal component analysis (PCoA) with corresponding PERMANOVA results to right of each graph. Symbol color represents individual cages. Stacked taxa bar plots, collapsed to the order level, detailing the top six most abundant orders present at 5 (**E**) and 10 (**F**) months. **I**, **J** Examination of order and genus level taxa previously identified in the C5aR1KO separate housing study demonstrated revealed that cohousing eliminated differences in abundance at both ages. *p < 0.05; **p < 0.01; ***p < 0.001. ANOVA statistics are available in Additional file 6: Table S1 while beta-diversity PERMANOVAs can be found in Additional file 6: Table S2. Additional details are available in Additional file 3

As animal housing (cage effects) contributed significantly to microbial diversity, we repeated the above experiment with a larger number of mice, but cohorts were generated and housed such that WT and/or ArcC5aR1+ animals had WT and/or Arc C5aR1KO cage mates. Fecal samples were collected at 5 and 10 months of age and sequenced using 16S Illumina primers targeting the V3 and V4 regions (Fig. 3A). Analysis was limited to animals with samples available at both 5 and 10 months of age. Main figure statistics are available in Additional file 6: Table S1 and Additional file 7: Table S2, with additional information available in Additional file 3.

At 5 months of age (during rapid accumulation of amyloid plaques), the cohousing of WT and Arc C5aR1+ and C5aR1KO mice prevented C5aR1KO-induced changes in alpha diversity (Fig. 3B). The lack of differences in alpha diversity between the groups persisted at 10 months of age (Fig. 3B). Furthermore, PERMANOVA analysis at each age demonstrated that there were no differences in the overall composition (beta-diversity) of the microbiome at 5 months or 10 months between the mice (Fig. 3C, D). This is reflected by the high degree of overlap between the groups in the PcoA plots for each age. Cage effects again accounted for a large portion (31–33%) of the observed variance at both 5 (*R*^2^ = 0.314, *p* = 0.691) and 10 months (*R*^2^ = 0.329, *p* = 0.549), however they were non-significant (Additional file 7: Table S2).

Relative abundance plots of the most abundant orders demonstrate similar abundances for each identified order across all genotypes, with little difference between 5 and 10 months of age (Fig. 3E, F). *Bacteroidales, Clostridiales*, and *Erysipelotrichales* were the top 3 most abundant taxa. No differences in *Erysipelotrichales, Clostridiales*, *Alistipes* or *Turicibacter* were observed among 5- or 10-month cohoused animals when analyzed by two-way ANOVA (Fig. 3G–J) (see Additional file 2 for three-way ANOVA results including age as a factor). Together, these data demonstrated that the impact of C5aR1 deletion on microbial composition is overcome by the cohousing of C5aR1KO and C5aR1-sufficient mice.

### Systemic inhibition of C5aR1 in singly housed adult wildtype or mouse models of AD does not alter the microbiome

To compare the effect of a global lifetime C5aR1 genetic ablation with pharmacological inhibition of C5aR1in adult mice, we explored the effects of systemic C5aR1 inhibition on the bacterial communities of the gut via treatment with the C5aR1 antagonist, PMX205, for 12 weeks, in wild-type mice and during the onset of AD pathology in AD model mice. Here, we explored the impact on the gut microbiome in two mouse models, Arctic and Tg2576, with treatment beginning at 2 and 12 months, respectively. As these two mouse models have different genetic backgrounds and have different timing and expression patterns of AD pathology, they provide the opportunity to determine if treatment with PMX205 yields similar results in different models of AD. Animals were singly housed for the duration of drug treatment. All two-way ANOVA statistics are available in Additional file 6: Table S1, beta-diversity statistics in Additional file 7: Table S2, with additional information available in Additional file 4.

**Fig. 4:**
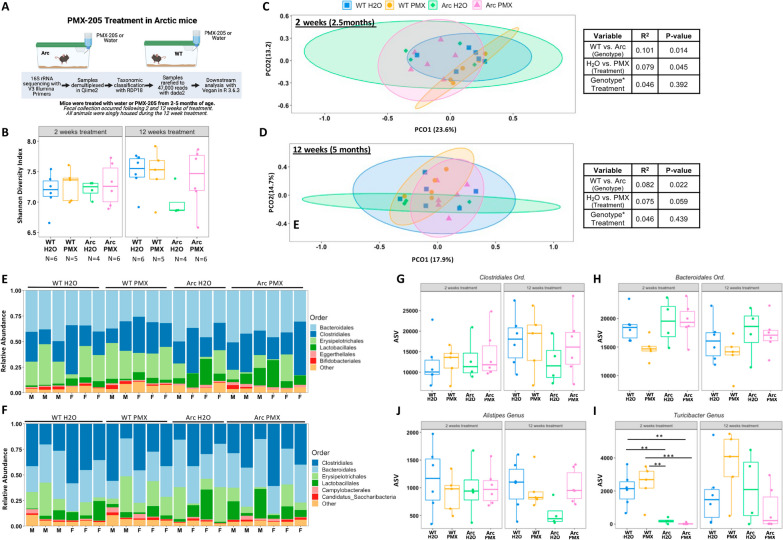
Treatment with PMX205 does not alter the microbiome of Arctic mice. **A** Schematic of the Arctic PMX205 study design. **B** Alpha diversity at 2 months of age, following 2 weeks of treatment with water or PMX205 or 5 months of age following 12 weeks of PMX205. **C** Beta-diversity, as measured by Bray–Curtis dissimilarities. Beta-diversity is presented as principal component analysis (PCoA) with corresponding PERMANOVA results to right of each graph that the microbiota composition was not altered due to the interaction of genotype and treatment at 2 (**C**) or 5 (**D**) months. Stacked taxa bar plots, collapsed to the order level, detailing the top six most abundant orders present following 2 (**E**) and 12 (**F**) weeks of treatment. **G**–**J** Specific order and genera were analyzed at each age by two-way ANOVA. **p < 0.01; ***p < 0.001 ANOVA statistics are available in Additional file 6: Table S1 while beta-diversity PERMANOVA can be found in Additional file 7: Table S2. Additional details are available in Additional file 4

Fecal samples from wildtype and Arc animals were collected following 2 and 12 weeks of treatment with or without PMX205 in the drinking water (Fig. 4A). No significant differences in either wildtype or Arc mice were observed in alpha diversity at either treatment time point when analyzed by two-way ANOVA (Fig. 4B). However, at 5 months, Arc-H_2_O mice displayed a trending decrease in their alpha diversity compared to WT-H_2_O mice that was partially recovered by treatment with PMX205 (*p* = 0.137, *t*-test between Arc-H_2_O and Arc-PMX after 12 weeks) (Additional file 8: Fig. S7D). Together, these results suggest Arctic mice had a smaller variety of species present in their fecal microbiome when separated from WT mice at an early age (2 mo, just as amyloid plaque accumulation is beginning), and that continued treatment with PMX205 may prevent or delay this decrease.

We next asked if treatment with PMX205 altered the beta-diversity of WT and Arc mice, as seen in the C5aR1KO animals with segregated housing. PERMANOVA analysis revealed there were significant effects of the Arc transgene at both 2- and 12-week time points (Fig. 4C, D; Additional file 7: Table S2). When all untreated mice (both WT and Arc) were compared to all treated mice (WT and Arc), inhibition of C5aR1 contributed slightly but significantly to the microbial composition after 2 weeks (*R*^2^ = 0.79, *p* = 0.045) (Fig. 4C), but there was only a trend for an effect after 12 weeks (*R*^2^ = 0.075, *p* = 0.059) of treatment (Fig. 4D). However, at no point during the study was the composition of the microbiome significantly influenced by the inhibition of C5aR1 within a genotype (Additional file 8: Fig. S7; Additional file 4). Thus, while there may be a small overall effect of PMX205 on beta-diversity, the lack of any significance in post hoc tests suggests that PMX205 has a minimal impact on microbial composition.

To further confirm the lack of changes in microbial composition due to PMX205 treatment, stacked taxa bar plots were graphed at the Order level. *Bacteroidales, Clostridiales,* and *Erysipelotrichales* were identified as the top 3 most abundant taxa at 2 weeks post-treatment (Fig. 4E). After 12 weeks of treatment, *Clostridiales* became the most prominent taxon across the groups followed by *Bacteroidales* (Fig. 4F) despite only trending changes in their overall abundance of these two taxa between 2 and 12 weeks (*p* = 0.094, *p* = 0.063, respectively). These changes appear to be an effect of age and not genotype or treatment, as no significant differences were observed between any groups when *Clostridiales* (Fig. 4G) or *Bacteroidales* (Fig. 4H) were examined at each individual time point (Fig. 4H). While the genotype or treatment of an animal did not influence *Alistipes* abundance*,* there was a trending interaction (*p* = 0.077) between the two variables, demonstrating a potential rescue of *Alistipes* abundance in Arc-PMX treated mice relative to untreated Arc (*p* = 0.157), but only at 5 m of age after 12 weeks of treatment (Fig. 4I). In contrast, Arc animals had significantly decreased abundances of *Turicibacter* at 2.5 mo after only 2 weeks of treatment, compared to WT mice (*p* < 0.01), regardless of treatment with the C5aR1 antagonist. However, by 12 weeks of treatment (5 m of age) the differences had dissipated. Thus, this 12-week treatment with PMX205 at the onset of AD pathology did not significantly alter the microbiome of WT or Arc mice. These results are consistent with those from the C5aR1KO cohousing study assessed at 5 months of age.

**Fig. 5:**
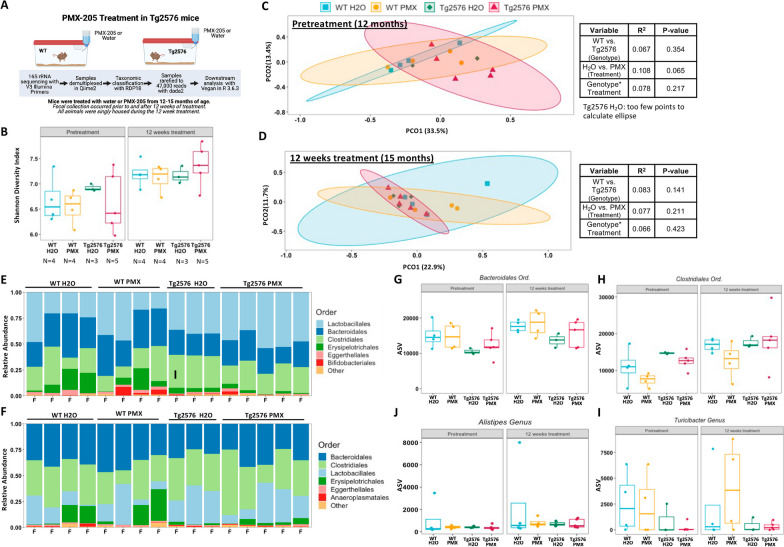
Treatment with PMX205 does not significantly alter the microbiome of Tg2576 mice. **A** Schematic of the Tg2576 PMX205 study design. Alpha diversity at **B** 12 months of age, prior to treatment with water or PMX205 or **C** 15 months of age following 12 weeks of treatment in Tg2576. Beta-diversity, as measured by Bray–Curtis dissimilarities revealed no significant difference between the interaction of genotype and treatment before (**C**) or after (**D**) 12 weeks of PMX205 treatment. Stacked taxa bar plots, collapsed to the order level, detailing the top six most abundant orders present before (**E**) and after (**F**) PMX205 treatment. **G**–**J** Examination of order and genus level taxa from the Arctic PMX205 study. ANOVA statistics are available in Table S1 while beta-diversity PERMANOVA can be found in Additional file 7: Table S2. Additional details are available in Additional file 4

A second mouse model of amyloidosis, Tg2576, was treated with PMX205 to determine if the results in Arc were specific to that model. Tg2576 mice begin to develop amyloid plaques at approximately 12 months of age, with pathology beginning in the cortex, rather than the hippocampus as observed in the Arc animals. As females develop more severe and earlier AD pathology than males, only female mice were used for this study. Fecal samples were collected immediately prior to the beginning of PMX205 treatment, at 12 months of age, and again following 12 weeks of treatment (Fig. 5A). The corresponding pathological, synaptic, and microglial changes for these mice are available in Gomez-Arboledas et al. [35].

As with the Arctic PMX205 study, no differences in alpha diversity were observed between any of the groups prior to or after 12 weeks of PMX205 treatment in the Tg2576 mice (Fig. 5B). The lack of differences in alpha diversity between WT and Tg2576 at both time points is consistent with previous work [8]. Examination of the beta-diversity yielded results that differed from the Arctic PMX205 study, as microbial composition was not affected by the APP transgene, PMX205 treatment, or their interactions, both prior to or after antagonist treatment (Fig. 5C, D; Table S2). Surprisingly, there was a trending effect in the group selected for PMX205 treatment (*p* = 0.065), indicating a unique microbiota composition prior to the actual initiation of the experiment (Fig. 5C left). However, the effect was small and likely due to how mice were separated for this experiment, as cagemates were not always assigned to different treatments.

Prior to treatment, the most abundant Orders were *Lactobacillales*, *Bacteroidales,* and *Clostridiales*, with *Lactobacillales* dominating, as demonstrated by the stacked taxa bar plot (Fig. 5E). However, by 12 weeks post-treatment, *Bacteroidales* became the most abundant bacteria, followed by *Clostridiales* and *Lactobacillales* (Fig. 5F). No changes in *Clostridiales* abundance were observed prior to or after PMX205 treatment (Fig. 5G). In contrast, Tg2576 animals had decreased levels of *Bacteroidales* regardless of what treatment they were assigned prior to the onset of treatment (*p* < 0.01), with a trending decrease observed in Tg2576 versus WT abundance after 12 weeks treatment (*p* = 0.065; Fig. 5H, Additional file 8: Fig S8). No differences in *Alistipes* or *Turicibacter* were observed (Fig. 5I, J).

## Discussion

The complement system is critical for controlling microbial infection, but is also a component of pathological inflammation. In targeting this cascade to mitigate damage due to inappropriate or excessive complement activation, it is important to also consider the impact of that modulation on the microbiome. Deletion or inhibition of various components of the complement cascade has shown promise in mouse models of Alzheimer’s disease, supportive of translation to trials in the clinic (reviewed in [2]). Here, the associations between the fecal microbiome and the complement system in mouse models of Alzheimer’s disease were assessed. The data show that (1) adult deletion of C1q has a subtle but statistically significant impact on the microbiome in the Arctic model of AD, but not in WT mice; (2) constitutive deletion of C5aR1 significantly altered the fecal microbiome of both WT and Arc mice when C5aR1-deficient mice were housed separately from C5aR1-sufficient counterparts; this alteration was not observed in cohoused C5aR1KO and C5aR1+ mice; and (3) treatment of two AD models with the C5aR1 inhibitor PMX205 had no effect on the fecal microbiome, despite these mice being singly housed for the duration of the treatment. While sample sizes in the later cases were not large, the comprehensive impact of complement on the AD microbiome across experiments supports these conclusions. Additionally, sex is known to contribute to microbiota composition in both health and AD [48, 49]. While both males and females were used in all Arctic mouse studies, the small sample sizes limited our ability to fully assess the impact of sex on the microbiome following complement inhibition in healthy and AD mice, although no sex differences were noted in other pathological or behavioral assessments in these Arctic mice [32, 33]. As such, sex-dependent changes may be masking changes in the microbiome that would only be revealed with a larger sample size.

While we did not observe any differences in C1q deficient vs sufficient WT mice, the fecal microbiota composition of Arctic C1q^−/−^ animals was significantly altered, suggesting that C1q affects the microbiome only when injury or challenge is present. Amyloid accumulation and damaged neurons are associated with induced expression of C1q in the brain [50] and amyloid in beta sheet fibrils as well as hyperphosphorylated tau, apoptotic cells and weak synapses provide complement pathway activators interacting with C1q (in C1) to activate the classical complement cascade. C1q deletion in Arctic mice increased *Alistipes* and decreased *Turicibacter* levels, both of which are also observed in AD [6, 11]. While this study does not explore the impact of these changes, it does suggest that C1q inhibition in AD may promote changes in microbiome associated with AD pathology, which may potentially diminish the effectiveness of anti-C1q therapeutics. Studies in zebrafish deficient in *irf8* demonstrated a reduced macrophage population resulting in reduced expression of C1q. The reduction of C1q was also correlated with the dysregulation of intestinal microbiota and outgrowth of rare bacterial species, postulating a role for C1q in the elimination of these microbes to shape the microbiome composition [51]. However, it remains to be determined if this is a direct result of C1q, a function of decreased macrophages, or an absence the *irf8* transcription factor, which is known to exert anti-microbial defenses [52]. A recent report in mice demonstrated that constitutive loss of C1q in macrophages did not alter the microbiome of young, healthy mice, yet macrophage C1q was critical for regulating gut motility, potentially by modulating enteric neurons [26]. Critically, both our globally WT C1q^−/−^ and Arctic C1q^−/−^ mice were C1q sufficient until 6 months of age, eliminating any potential confounds of C1q deficiency during development and suggesting that C1q contributes to maintaining eubiosis in the presence of chronic systemic inflammation, as no divergence from WT was observed of the Arctic microbiome when C1q remained present. Whether this is via interactions with enteric neurons or modulation of the immune response, which in turn shape the microbiome remains unknown. Surprisingly, in this study, which required oral gavage to induce deletion of C1q in adult mice, we observed an increase in the alpha diversity of Arctic mice relative to WT mice, regardless of C1q deletion. As previous literature has demonstrated an age-related decline in the alpha diversity occurs in various AD models [8, 11, 53], this result is unexpected. However, as no untreated mice were included in this analysis, it is possible the vehicle and tamoxifen gavages negatively impacted the WT but not Arctic alpha diversity without disrupting the composition of the microbiomes.

When C5aR1KO mice are housed apart from their C5aR1-sufficient counterparts, the deletion of C5aR1 significantly reduces the diversity microbes, resulting in an altered composition of the fecal microbiome in both healthy and AD mice. In the oral microbiota, *P. gingivalis* which has been repeatedly linked to AD [54–56], is able to evade human and mouse neutrophils and macrophages by initiating crosstalk between C5aR1 and TLR2 by bypassing host protective MyD88 signaling pathways and instead inducing a pro-inflammatory TLR2–TIRAP–PI3K pathway, in which phagocytosis is inhibited [57–59]. Furthermore, gingipain-1, a proteinase derived *from P. gingivalis,* induces cleavage of C5 and generation of functional C5a [60]. In addition, deletion of C5aR1 reduced diversity and altered the composition of the cutaneous microbiome [29], and in the gut, deletion of C5aR1 was associated with decreased levels of *Lactobacillus spp.*, a potentially protective bacterium [61]. Furthermore, C5aR1 was shown to modulate intestinal tight junctions in an IgE-mediated food allergy by indirectly modulating the histamine-mediated opening of endothelial tight junctions [62]. Taken together, C5aR1 can influence the host–microbiome interface by modulating the local and systemic innate immune response, and/or by increasing the permeability of, and thus microbial entry through, the intestinal barrier. Additionally, microbes may be able to subvert the immune system by commandeering C5a-C5aR1 signaling.

In this study, the impact of C5aR1 deletion on microbial composition was overcome by the cohousing of C5aR1KO and C5aR1+ mice whether WT or Arctic, but it remains possible that specific species of bacteria, undetected in the current analyses, were altered in cohoused C5aR1KO mice, but overall large-scale microbiota changes were prevented. Cage effects are a well-known occurrence in microbiota studies, including those examining the microbiome composition of AD models or other innate immune deletions [11, 63–66]. Unsurprisingly, PERMANOVA results repeatedly identified the largest proportion of the observed variability in our study was due to the specific cage the animal resided in, whereas the effects of genotype and complement ablation resulted in relatively small shifts in microbiome composition.

We have previously demonstrated constitutive deletion of C5aR1 delays microglial polarization and improves cognition performance in Arc mice housed according to our separately housed paradigm (C5aR1+ vs C5aR1KO), without changing amyloid plaque density [33]. While the microbiomes of ArcC5aR1+ and ArcC5aRKO mice were indeed different from one another, it is unlikely (but not impossible) that the differences in the microbiome contributed to long-term memory recovery in Arc C5aR1KO as the WT and Arc, where behavioral differences were detected, had similar microbiomes. A future study assessing behavior of cohoused ArcC5aR1+ and ArcC5aR1KO mice together would be required to further exclude the microbiome as a contributor to disease phenotype.

However, importantly, there was no effect of PMX205 treatment in WT (C57B6/J) or Arctic or WT (C57/SJL) and Tg2576 mice, demonstrating adult inhibition of C5aR1 does not impact the fecal microbiome. While studies have demonstrated conflicting evidence of dysbiosis of Tg2576 mice at 15 months of age [8, 10], the Tenner lab has previously demonstrated treatment with PMX205 in Tg2576 mice from 12 to 15 months of age significantly improves synaptic protein levels while reducing plaque burden, dystrophic neurites, and shifting microglial towards a neuroprotective phenotype [34, 35]. Thus, in our current studies (utilizing fecal samples from the mice examined in [35]), it is evident that the underlying improvement of AD pathology in Tg2576 mice was not due to any large-scale changes in the microbiome.

## Conclusion

It is critical that researchers consider potential impacts on the microbiome when designing their studies. This study demonstrates an influence of C1q and C5aR1 on the microbiome on two AD mouse models. Deletion of C1q results in changes in the Arctic fecal microbiome, but not in WT mice. The difference induced by the lack of C5aR1 in both WT and Arctic mice was, however, overcome when animals were cohoused with C5aR1-sufficient mice, and no differences were detected when C5aR1 signaling was pharmacologically inhibited in either Arc or Tg2576 mice. These data further suggest that the functional protection seen in AD mouse models by inhibition of C1q or C5aR1 is likely not due to detectable changes in the microbiomes of these animals. These studies also underscore the importance of considering and reporting housing methodologies in microbiome studies.

### Supplementary Information

**Additional file 1.** C1q RosaCre statistics.

**Additional file 2.** C5aR1KO separate housing statistics.

**Additional file 3.** C5aR1KO cohousing statistics.

**Additional file 4.** Arctic and Tg2576 PMX205 statistics.

**Additional file 5.** Supplemental Methods.

**Additional file 6: Table S1.** Two-way ANOVA results for main figures.

**Additional file 7: Table S2. **PERMANOVA results for main figures.

**Additional file 8:** Supplemental Figures. **Figure S1**. C1q is not detectable in plasma or hippocampus 6 months after tamoxifen treatment in RosaCre+ mice. **Figure S2. **The presence of the RosaCre transgene does not affect the alpha or beta diversity in vehicle treated WT C1qa^FL/FL^ and Arctic C1qa^FL/FL^ mice. **Figure S3.** Tamoxifen treatment did not alter the microbial diversity in WT and Arctic C1qa^FL/FL^ mice lacking RosaCre. **Figure S4.** Deletion of C1qa in Arctic but not WT mice alters beta-diversity. **Figure S5.** Arctic mice have an increased alpha diversity independent of C1q deletion. **Figure S6.** Tamoxifen treatment of WT and Arctic mice with and without RosaCre demonstrates changes due to Arctic genotype and C1q deletion. **Figure S7. **PMX205 does not alter the microbiome of WT or Arctic mice. **Figure S8.** Bacteroidales levels increase with age in Tg2576 mice but remain lower than those in WT mice.

## Data Availability

Data used in this study are available on the NCBI Sequence Read Archive (SRA) under BioProject accession number PRJNA957778.

## Abbreviations

AD: Alzheimer’s disease
APP: Amyloid precursor protein
Arc: Arctic48
ASV: Amplicon sequence variant
WT: Wild type
