# Supplementary material for: Influence of complement protein C1q or complement receptor C5aR1 on gut microbiota composition in wildtype and Alzheimer’s mouse models
Source: J Neuroinflammation. 2023 Sep 19;20:211. doi: 10.1186/s12974-023-02885-9 (PMC10507976; doi:10.1186/s12974-023-02885-9)
Supplement: Supplementary file 5 — Additional file 5. Supplemental Methods. [file 12974_2023_2885_MOESM5_ESM.docx]

**Supplemental Methods**

**Tissue Collection:**  Brain tissue was processed as previously described (1). Briefly, mice were anesthetized with isoflurane and perfused with PBS. Brains were collected. Half of the brain was fixed for 24 hours with 4% paraformaldehyde and hereafter stored in PBS/0.02% sodium azide at 4°C until use.  The remaining half was dissected into hippocampus and cortex and immediately frozen on dry ice and stored at -80°C until needed.

**Immunohistochemistry**: Immunostaining procedures were performed as described (2) . Briefly, 40 um coronal sections of brain, were incubated overnight at 4°C with primary antibody anti mouse C1q (rabbit monoclonal, clone 27.1) tissue culture supernatant (3). C1q was detected with Alexa-555 (Invitrogen, Carlsbad, CA) followed by counterstaining with Thioflavin-S (Sigma-Aldrich, #T1892). Briefly, sections were incubated in 0.1% ThioS diluted in MilliQH_2_O for 10 minutes and washed 2X with 50% ethanol followed by PBS. The images were acquired with a ZEISS Axio ScanZ1 Digital Slide Scanner at 10x magnification (Zeiss, Thornwood, NY). Images were acquired with the same exposure times and camera settings when comparing the same brain area in the different genotypes. For quantitative analysis of C1q in the molecular layer of hippocampus, images were analyzed using ImageJ software. Five regions of interest (ROI) (squares) were defined randomly within the molecular layer area and the mean pixel intensity per ROI was determined. The mean intensity of each animal was obtained by averaging all ROI mean intensities in the sections studied. ThioS field area percent was assessed using IMARIS as previously described (4). One way ANOVA statistical analysis was used to assess the differences in ThioS percent field area(Prism 9.0, GraphPad).

**Western Blot**: Each half brain or hippocampus was pulverized, aliquoted and stored at -80°C. Brain powder was solubilized by homogenization in 10 volumes of Tris-buffered saline (TBS, pH 7.4) containing protease inhibitor cocktail solution (Complete mini, Roche), 1mM EDTA and 1% SDS using a motor pestle for 5 seconds twice on ice. After centrifugation at 18,400xg for 30 minutes at 4°C, protein concentration in the supernatants was determined with a BCA protein assay (Pierce, Rockford, IL). Thirty µg of brain protein or 1ul of plasma were subjected to SDS-polyacrylamide gel electrophoresis (1%) under reducing conditions. Gels were transferred at 4°C in Tris-glycine, 0.02% SDS, and 10% methanol transfer buffer onto polyvinylidene difluoride (PVDF, Immobilon-P, Millipore) membrane at 300 mA for 2 hours. The PVDF membrane was blocked one hour at RT in 5% nonfat dry milk in TBS- 0.1% Tween. Rabbit anti-mouse C1q (1151) (5) or anti-ß-actin (Sigma) in either 5 or 3% milk, respectively, was applied overnight at 4˚C. After washing, HRP-conjugated secondary antibodies, diluted 1:5000 (Jackson Labs), were added and incubated for 1 hour at RT. The blots were developed using ECL 2 (Pierce) for C1q and ECL (Pierce) for ß-actin, and analyzed using a BioRad ChemiDoc image system and the Image J software as described (6).

**References**

1. Fonseca MI, Ager RR, Chu SH, Yazan O, Sanderson SD, LaFerla FM, Taylor SM, Woodruff TM, Tenner AJ. 2009. Treatment with a C5aR antagonist decreases pathology and enhances behavioral performance in murine models of Alzheimer's disease. J Immunol 183:1375-83.

2. Fonseca MI, Chu SH, Hernandez MX, Fang MJ, Modarresi L, Selvan P, MacGregor GR, Tenner AJ. 2017. Cell-specific deletion of C1qa identifies microglia as the dominant source of C1q in mouse brain. J Neuroinflammation 14:48.

3. Stephan AH, Madison DV, Mateos JM, Fraser DA, Lovelett EA, Coutellier L, Kim L, Tsai HH, Huang EJ, Rowitch DH, Berns DS, Tenner AJ, Shamloo M, Barres BA. 2013. A dramatic increase of C1q protein in the CNS during normal aging. JNeurosci 33:13460-13474.

4. Gomez-Arboledas A, Carvalho K, Balderrama-Gutierrez G, Chu SH, Liang HY, Schartz ND, Selvan P, Petrisko TJ, Pan MA, Mortazavi A, Tenner AJ. 2022. C5aR1 antagonism alters microglial polarization and mitigates disease progression in a mouse model of Alzheimer's disease. Acta Neuropathol Commun 10:116.

5. Huang J, Kim LJ, Mealey R, Marsh HC, Jr., Zhang Y, Tenner AJ, Connolly ES, Jr., Pinsky DJ. 1999. Neuronal protection in stroke by an sLex-glycosylated complement inhibitory protein. Science 285:595-599.

6. Khoury MK, Parker I, Aswad DW. 2010. Acquisition of chemiluminescent signals from immunoblots with a digital single-lens reflex camera. AnalBiochem 397:129-131.
