# Supplementary material for: Influence of complement protein C1q or complement receptor C5aR1 on gut microbiota composition in wildtype and Alzheimer’s mouse models
Source: J Neuroinflammation. 2023 Sep 19;20:211. doi: 10.1186/s12974-023-02885-9 (PMC10507976; doi:10.1186/s12974-023-02885-9)
Supplement: Supplementary file 8 — Additional file 8: Supplemental Figures. Figure S1. C1q is not detectable in plasma or hippocampus 6 months after tamoxifen treatment in RosaCre+ mice. Figure S2. The presence of the RosaCre transgene does not affect the alpha or beta diversity in vehicle treated WT C1qaFL/FL and Arctic C1qaFL/FL mice. Figure S3. Tamoxifen treatment did not alter the microbial diversity in WT and Arctic C1qaFL/FL mice lacking RosaCre. Figure S4. Deletion of C1qa in Arctic but not WT mice alters beta-diversity. Figure S5. Arctic mice have an increased alpha diversity independent of C1q deletion. Figure S6. Tamoxifen treatment of WT and Arctic mice with and without RosaCre demonstrates changes due to Arctic genotype and C1q deletion. Figure S7. PMX205 does not alter the microbiome of WT or Arctic mice. Figure S8. Bacteroidales levels increase with age in Tg2576 mice but remain lower than those in WT mice. [file 12974_2023_2885_MOESM8_ESM.docx]

**Supplemental Figures:**

**
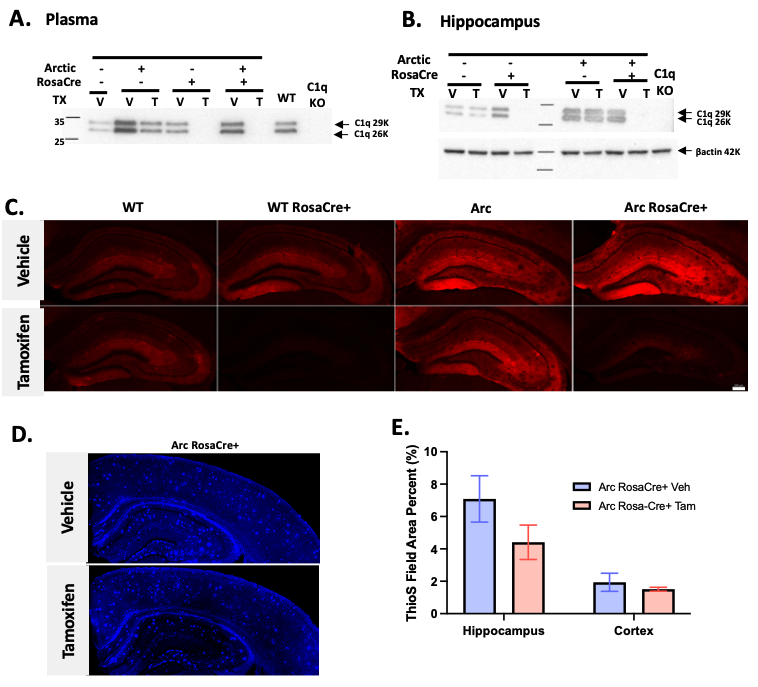
**

**Figure S1: C1q is not detectable in plasma or hippocampus 6 months after tamoxifen treatment in RosaCre+ mice.** Western blot of C1q in ***(A)*** plasma (1ul per lane) or ***(B)*** hippocampus (30 ug per lane) of 12.5 months old WT RosaCre-, WT RosaCre+, Arctic RosaCre- and Arctic RosaCre+ mice, 6m after vehicle (V) or tamoxifen (T) treatment. Wildtype (WT) and C1q knockout (C1qKO) plasma or hippocampus served as positive and negative controls, respectively. Representative of 2-5 animals/treatment/genotype. ***(C)*** C1q immunostaining (red) of hippocampus from 12.5 months old mice after vehicle (top row) and tamoxifen (bottom row) treatment. Representative images of 3-11 animals per genotype per treatment. Acquisition time and camera gain are identical for each panel. Scale bar: 200um. ***(D)*** Thioflavin S immunostaining (blue) and ***(E)*** percent field area quantification of Thioflavin S amyloid staining in hippocampus and cortex in Arctic RosaCre+ mice after vehicle (left) or tamoxifen (right) treatment. Representative images of 3 animals/genotype/treatment. HP, hippocampus; CTX cortex. P-value of HP =0.18 (t-test). Both males and females were used in the western blots; however no differences between sexes have been observed.

**
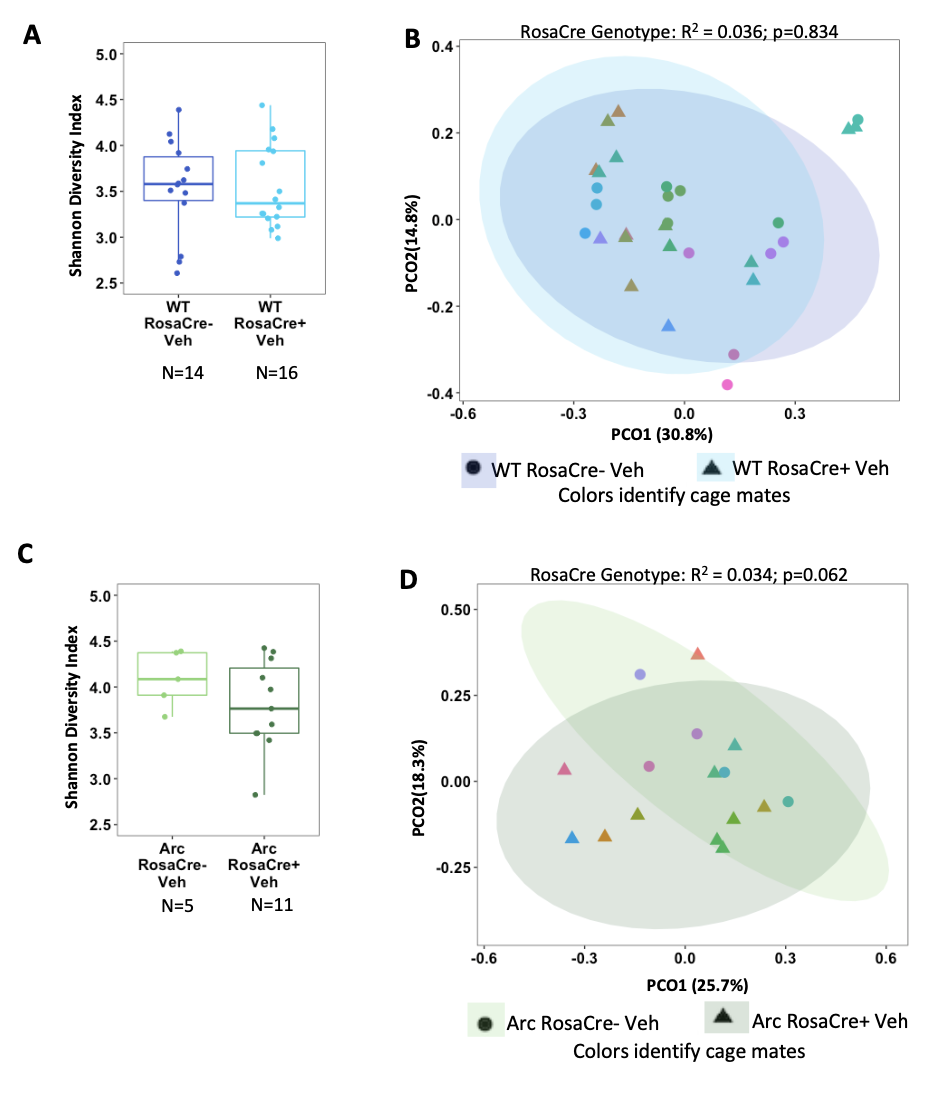
**

**Figure S2: The presence of the RosaCre transgene does not affect the alpha or beta diversity in vehicle treated WT C1qa^FL/FL^ and Arctic C1qa^FL/FL^ mice.**  Alpha diversity by the Shannon Diversity Index of WT (***A***) and Arc *(****C***) vehicle treated mice with and without RosaCre transgene. Beta diversity PCOA of Bray-Curtis Dissimilarity of WT (***B***) and Arc (***D***) vehicle treated mice with and without RosaCre transgene. Single factor fixed PERMANOVA for RosaCre genotype. All mice are C1qa^FL/FL^.

**
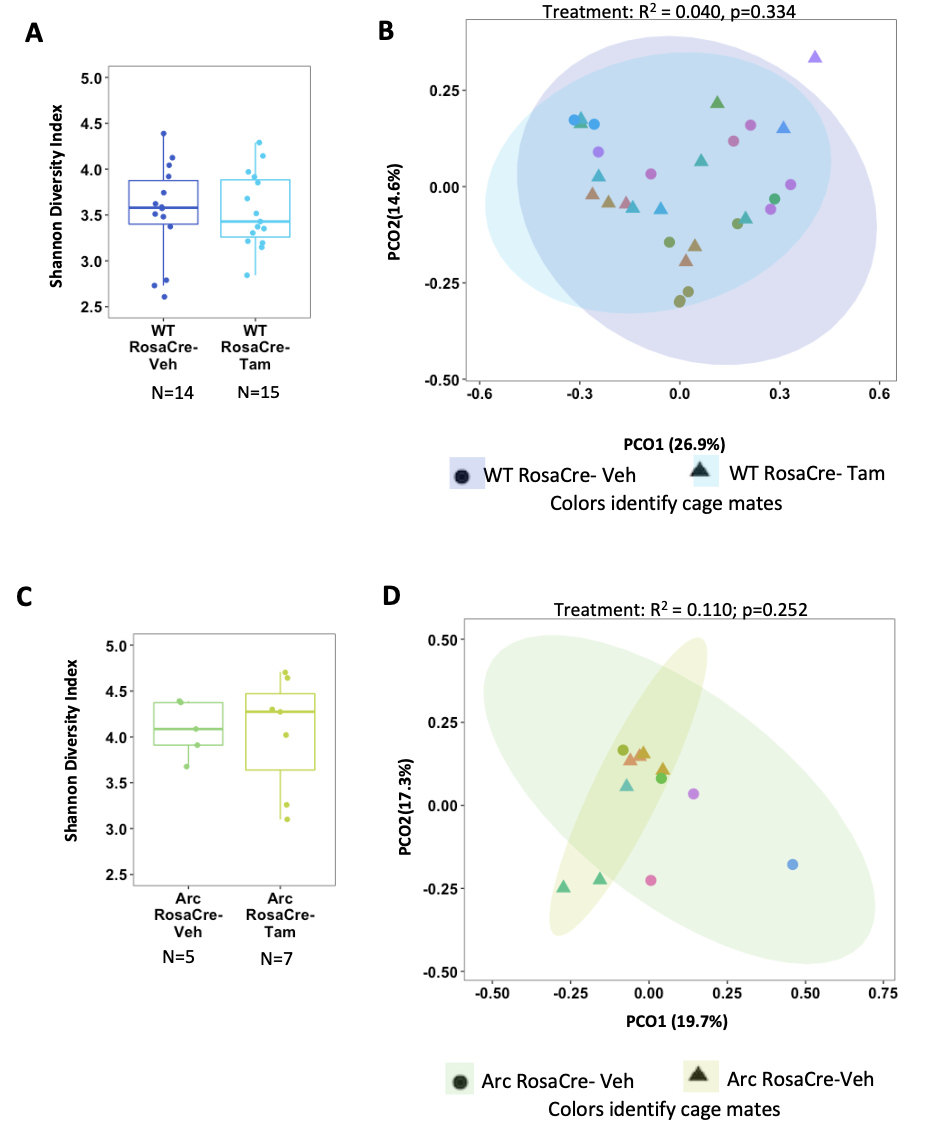
Figure S3: Tamoxifen treatment did not alter the microbial diversity in WT and Arctic C1qa^FL/FL^ mice lacking RosaCre. *A.*** The alpha diversity of WT RosaCre- mice was not affected by tamoxifen treatment. ***B.*** Beta diversity by Bray-Curtis Dissimilarity of WT animals revealed no effect of tamoxifen. ***C.*** Alpha diversity by Shannon Diversity Index of vehicle and tamoxifen treated Arctic RosaCre- mice. ***D.*** Analysis by Bray-Curtis Dissimilarity demonstrated no effect on beta diversity of tamoxifen on Arctic RosaCre- mice. Single factor fixed PERMANOVA value for treatment shown. All mice are C1qa^FL/FL^.

**
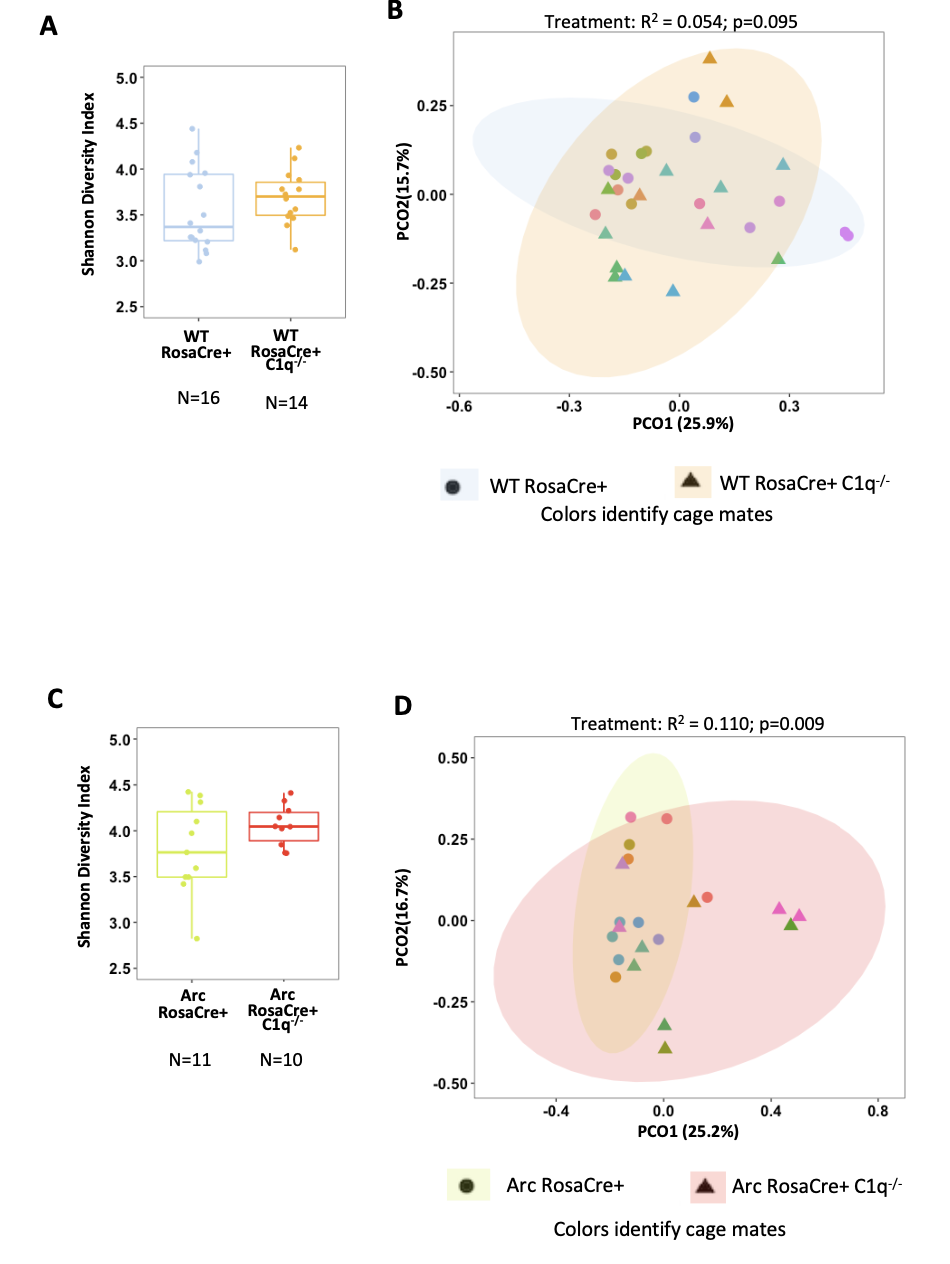
**

**Figure S4: Deletion of C1qa in Arctic but not WT mice alters beta-diversity *A.*** Alpha diversity and ***B.*** Beta diversity by Bray-Curtis Dissimilarity of WT RosaCre+ mice with or without C1q deletion ***C.*** Alpha diversity and ***D.*** Beta diversity by Bray-Curtis Dissimilarity of ArcRosaCre+ mice with or without C1q deletion PERMANOVAs were calculated as single fixed factor for treatment. All mice are C1qa^FL/FL^.

**
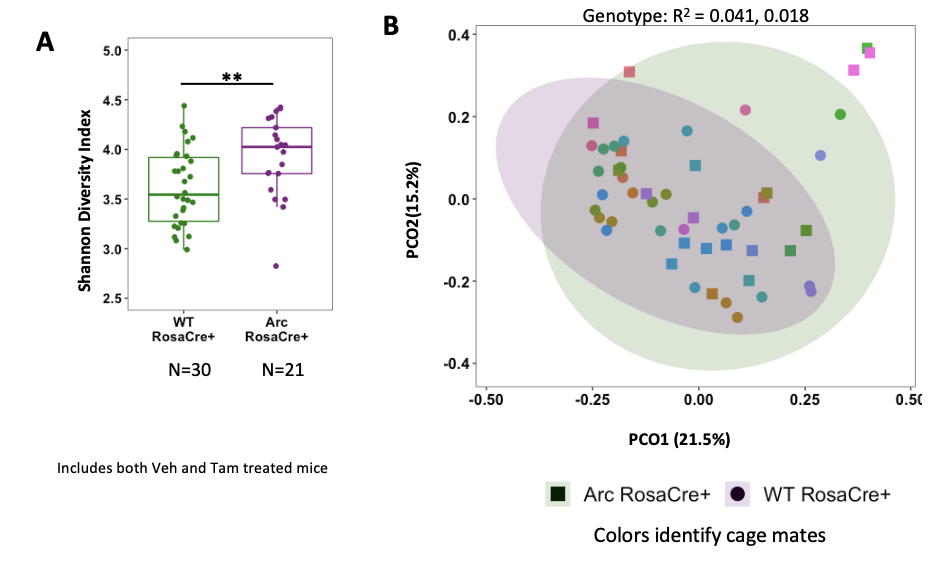
Figure S5.** **Arctic mice have an increased alpha diversity independent of C1q deletion.** ***A.*** Shannon diversity index of all WT and Arctic mice expressing RosaCre, regardless of vehicle or tamoxifen treatment ***B.*** Bray-Curtis Dissimilarity of all WT RosaCre+ mice or all RosaCre+ Arctic mice, regardless of treatment and graphed as a PCoA plot demonstrates that the microbiome of ArcRosaCre+ mice is significantly different from their WT counterparts. All mice are C1qa^FL/FL^. ** p<0.01.

**
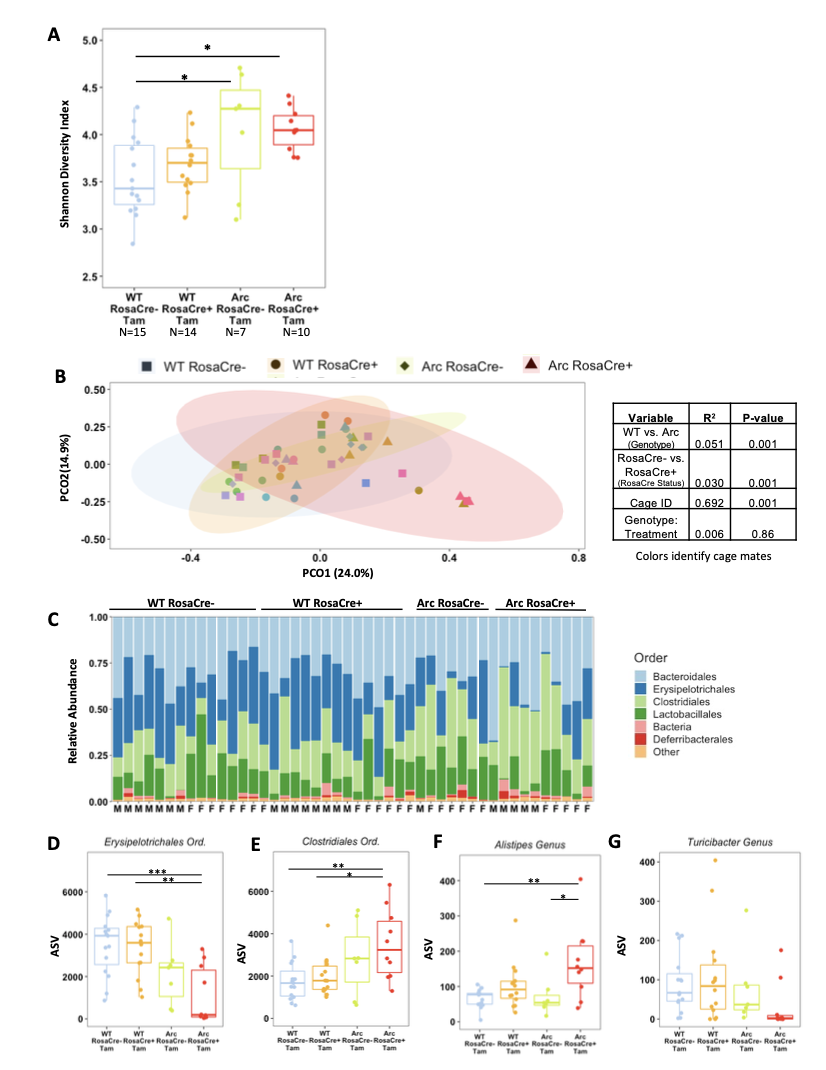
**

**Figure S6: Tamoxifen treatment of WT and Arctic mice with and without RosaCre demonstrates changes due to Arctic genotype and C1q deletion. *A.*** Alpha diversity between both WT and Arctic animals with and without the RosaCre transgene in tamoxifen treated mice shows effect of Arctic genotype at 12.5 mo of age. ***B.*** PCoA plot of beta diversity calculated by Bray-Curtis Dissimilarity for tamoxifen treated WT and Arctic animals with and without the RosaCre transgene. ***C.*** Relative abundance of Order taxa in tamoxifen-treated animals. ***D-G.*** Comparison of specific orders and genera in tamoxifen-treated animals. All mice are C1qa^FL/FL^. *p<0.05; ** p<0.01; ***p<0.001.

**
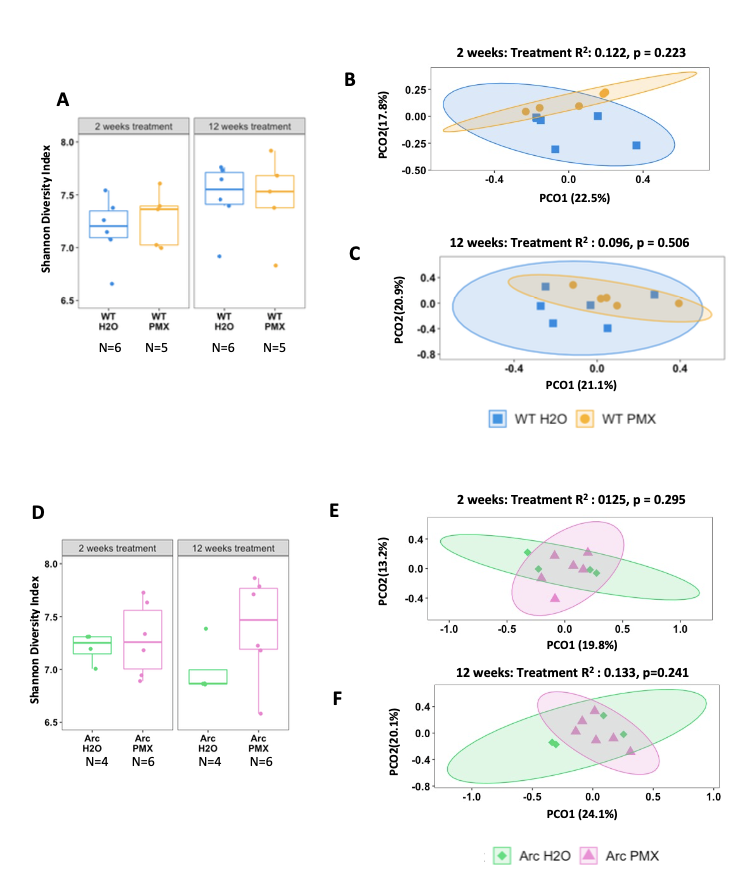
**

**Figure S7: PMX205 does not alter the microbiome of WT or Arctic mice. *A.*** PMX205 did not alter the alpha of WT mice after 2 or 12 weeks of treatment. ***B.*** Beta diversity by Bray-Curtis Dissimilarity demonstrates no effect of PMX205 treatment after 2 ***(B)*** or 12 ***(C)*** weeks*.* ***D.*** PMX205 did not affected the alpha diversity of Arctic mice following 2 or 12 weeks of treatment*.*  Beta diversity by Bray-Curtis Dissimilarity was not affected by 2 ***(E)*** or 12 ***(F)*** weeks of PMX205 treatment.

**
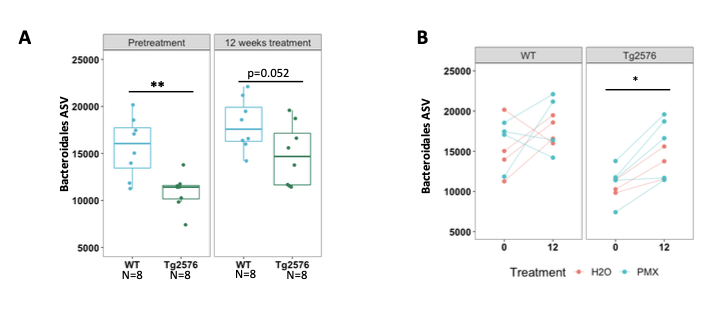
**

**Figure S8: Bacteroidales levels increase with age in Tg2576 mice but remain lower than those in WT mice.** ***A.*** Abundances of the order Bacteroidales in WT and Tg2576 mice, regardless of treatment, at the start of the study (12m of age) and at the conclusion of the study 12 weeks later (15m of age) demonstrated that Tg2576 mice had reduced levels of *Bacteroidales* at both ages compared to WT mice. ***B.*** Within-animal changes of *Bacteroidales* abundances between start and conclusion of the study demonstrated that, despite an increased abundance in *Bacteroidale*s from 12 to 15m of age in Tg2576 mice, these AD mice retained an almost significant decrease in *Bacteroidale*s compared to their WT counterparts.
